# Comprehensive list of SUMO targets in *Caenorhabditis elegans* and its implication for evolutionary conservation of SUMO signaling

**DOI:** 10.1038/s41598-018-19424-9

**Published:** 2018-01-18

**Authors:** Krzysztof Drabikowski, Jacqueline Ferralli, Michal Kistowski, Jacek Oledzki, Michal Dadlez, Ruth Chiquet-Ehrismann

**Affiliations:** 1 Institute of Biochemistry and Biophysics, Polish Academy of Science, Warszawa, Poland; 2 Friedrich Miescher Institute for Biomedical Research, Department of Mechanisms of Cancer, Maulbeerstrasse 66, CH-4058 Basel, Switzerland; 3 Institute of Genetics and Biotechnology, Biology Department, Warsaw University, Warszawa, Poland; 4 University of Basel, Faculty of Sciences, CH-4056 Basel, Switzerland

## Abstract

Post-translational modification by small ubiquitin-related modifier (SUMO) is a key regulator of cell physiology, modulating protein-protein and protein-DNA interactions. Recently, SUMO modifications were postulated to be involved in response to various stress stimuli. We aimed to identify the near complete set of proteins modified by SUMO and the dynamics of the modification in stress conditions in the higher eukaryote, *Caenorhabditis elegans*. We identified 874 proteins modified by SUMO in the worm. We have analyzed the SUMO modification in stress conditions including heat shock, DNA damage, arsenite induced cellular stress, ER and osmotic stress. In all these conditions the global levels of SUMOylation was significantly increased. These results show the evolutionary conservation of SUMO modifications in reaction to stress. Our analysis showed that SUMO targets are highly conserved throughout species. By comparing the SUMO targets among species, we approximated the total number of proteins modified in a given proteome to be at least 15–20%. We developed a web server designed for convenient prediction of potential SUMO modification based on experimental evidences in other species.

## Introduction

Small ubiquitin-related modifier (SUMO) proteins are covalently and reversibly coupled to intracellular protein targets, modulating protein-protein and protein-DNA interactions^1^. The post-translational protein modification by SUMO is achieved by similar mechanism as ubiquitination by a set of homologous but distinct enzymes^2^. The SUMO activation enzyme E1 (Aos1/Uba2 dimer) forms, in an ATP dependent manner, a thioester bond between its catalytical cysteine and the C-terminal carboxyl group of SUMO. Next, SUMO is transferred to the cysteine of the SUMO E2 conjugating enzyme, Ubc9. Finally, SUMO is transferred to the ε-group of lysine of the target protein, forming an isopeptide bond. This step is often achieved by the help of E3 SUMO ligase such as PIAS and RanBP2^3^. The SUMO moiety is removed from the target proteins by SUMO isopeptidases from the SENP family^4^. Modification by SUMO regulates various aspects of cell physiology including cell cycle, DNA replication and repair, transcription, epigenetic gene silencing, intracellular trafficking and cell signaling^5^. Despite the vast implications of SUMO in various cellular processes, only few enzymes mediating SUMOylation are known. In comparison, the ubiquitin conjugation system involves almost thousand proteins. Recent work suggested that SUMOylation specificity comes from spatiotemporal determinants and thus, such a big number of proteins, as in case of ubiquitination, is not necessary^6^.

The variety and number of cellular processes regulated by the post-translational modification by SUMO is comparable to processes regulated by phosphorylation and ubiquitination. Several SUMO targets belong to pathways involved in cancer and neurodegenerative diseases^7^. For example, increased levels of UBC-9, the SUMO conjugating enzyme, result in tumor formation^8^. In several tumors the levels of the SUMO E3 ligase PIAS are elevated^9^. SUMO modification regulates the protein levels and activity of tumor suppressor proteins, for example p53, p63, p73^10^ and MDM2^11^. Moreover, SUMO regulates the protein levels of huntingtin, amyloid precursor protein, DJ-1, ataxin-1, tau and superoxide dismutase^12^. Thus, SUMO attachment to disease related proteins is discussed as a potential therapeutic target^13^.

Post-translation modification by SUMO was shown to be influenced under stress conditions. SUMOylation increases in response to heat shock in human cells^14^, *Drosophila* embryos^15^ and *Arabidopsis*^16^. Ischemia, the cold shock, also increases global levels of SUMOylation in neurons^17^. Furthermore, SUMOylation is increased in the re-oxidation phase of oxygen/glucose deprivation, a model for ischemia^18^. In yeast SUMOylation increases in response to DNA damage^6^. Protein modification by SUMO conjugation machinery was shown to be directly regulated by the redox state of the cell^19,20^. Along the same lines, hydrogen peroxide treatment increased SUMOylation by SUMO2/3 but not via SUMO1^21^. On the other hand, SUMOylation decreased upon ER stress in *C. elegans*^22^. Furthermore, SUMO isopeptidases have been shown to be inactivated by heat^23^.

Initially, research was focused on the functions of SUMO modification in the nucleus, for example chromatin remodeling, genome stability and RNA. Recently, involvement of SUMO in non-nuclear processes is emerging such as in protein folding, lipogenesis, autophagy^24^, membrane transport and proteostasis^25^. Research in *C. elegans* revealed functions of SUMO in intermediate filament assembly^26^, ER stress^22^, chromosome assembly^27^ and assembly of adherens junctions to actin cytoskeleton^28^.

Recently, several whole proteome studies increased our knowledge about SUMO targets in human cells^29–33^. For recent compilation of SUMO targets in human cells please refer to a review by Henriks *et al*.^33^. However, SUMOylation is a very dynamic process which depends on the cell type, cell cycle stage and interactions of an organism with its environment. Thus, we investigated the targets of SUMO modification at the level of an entire organism, the nematode *Caenorhabditis elegans. C. elegans* is a complex higher eukaryote, but in contrast to vertebrates, contains only one SUMO gene. This gene is essential and needs to confer all functions of SUMO modifications in a higher organism. Our study identified a comprehensive list of SUMO targets. Interestingly, we show that those targets are remarkably evolutionary conserved. Thus, we propose a novel bioinformatics approach to predict SUMO modification based on experimental data of other species.

## Results

### SUMO is expressed throughout life of *C. elegans*

**Figure 1:**
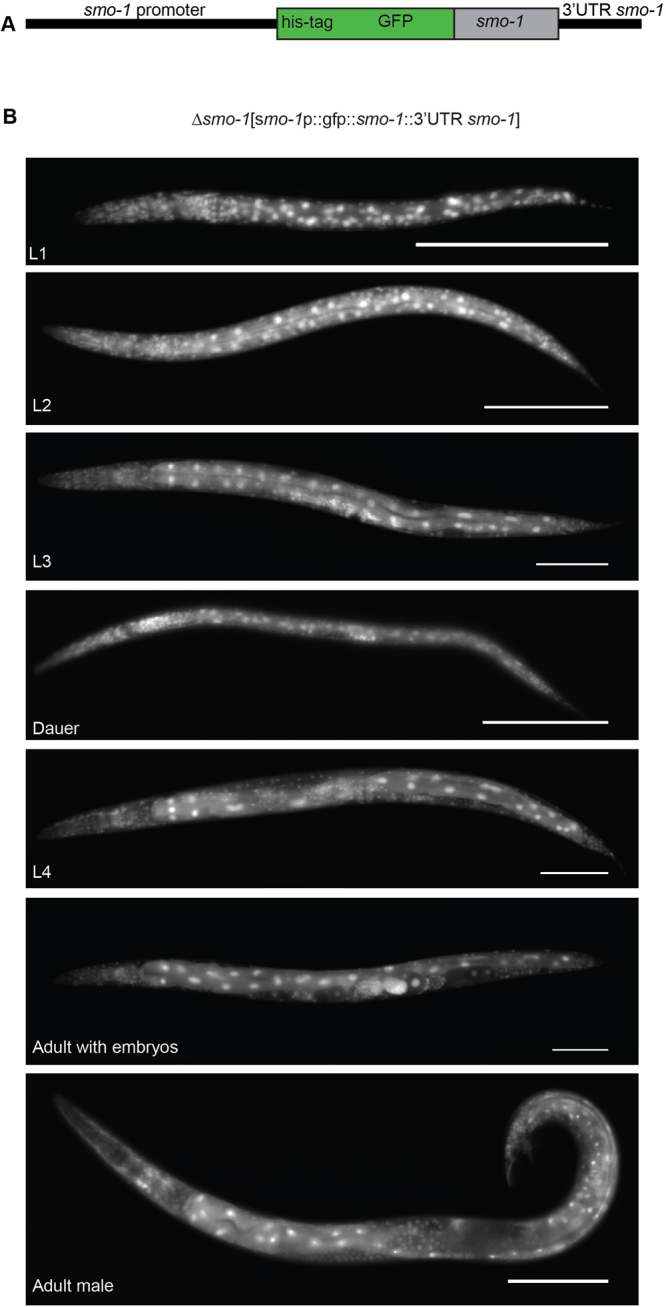
SUMO is expressed throughout development and in all cells in *C. elegans*. (**A**) Schema of translational fusion of HIS-tagged GFP with *smo-1* gene under the control of the endogenous *smo-1* promoter and including the endogenous 3′-UTR. (**B**) Fluorescent images of different developmental stages (L1, L2, L3, dauer, L4, adult hermaphrodite, adult male) of *C. elegans* stably expressing HIS-tagged GFP::*smo-1* construct in RU86. Anterior is to the left, ventral is to the bottom. Scale bar 100 µm.

**Figure 2:**
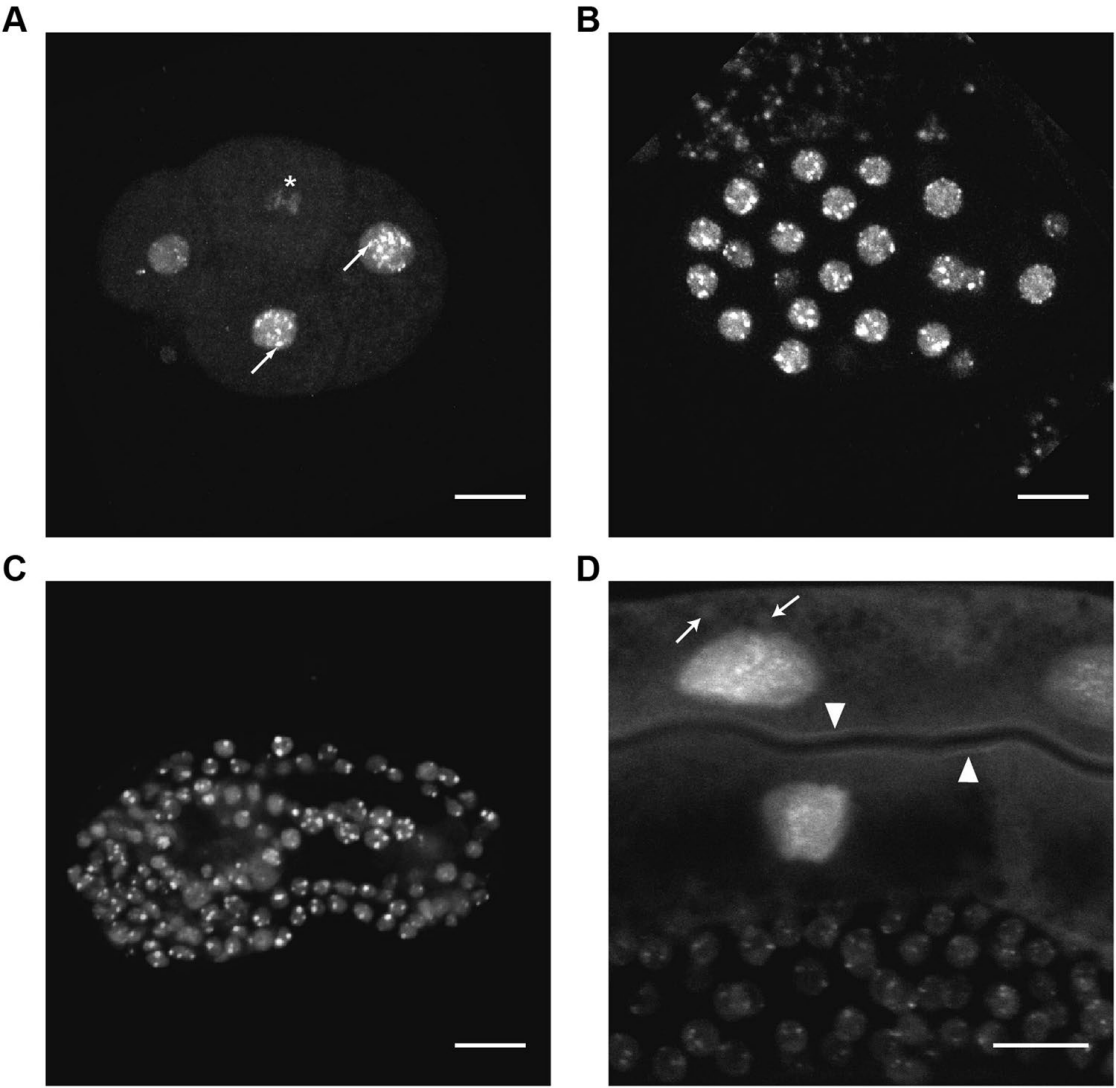
SUMO expression is enriched in the nucleus. (**A–D**) Stacked confocal images of subcellular localization of HIS-tagged GFP::*smo-1* in RU86. (**A**) 6-cell stage embryo. Arrow, nuclear speckles. Star, mitotic chromosomes (**B**) Embryo at gastrulation stage. (**C**) Comma stage embryo. (**D**) Zoom in into adult worm showing expression in the intestine and (upper part of image) and germline (lower part of image). Arrow, vesicles. Arrowhead, apical membrane. Scale bar 10 µm.

In *C. elegans* only one gene is coding for SUMO, *smo-1*. Deletion of *smo-1* is lethal. To analyze the posttranslational modification by SUMO in *C. elegans*, we generated a worm strain carrying a transgene composed of a N-terminally fused 8His-GFP to the worm SUMO gene (*smo-1*), called herein SUMO-GFP (Fig. 1A). The SUMO-GFP transgene rescues the lethal *smo-1* gene deletion in the VC186 strain. The SUMO-GFP fusion was expressed in all cells, including germ cells where the transgenes are often silenced, and throughout all developmental stages (Fig. 1B). The SUMO-GFP protein is localized predominantly in the nucleus but the cytoplasmic expression is also detectable, especially in the gut cells (Fig. 2). In the nucleus the protein is localized into speckles (Fig. 2A, arrows) or label dividing chromosomes (Fig. 2A, star). In the cytoplasm of gut cells SUMO-GFP can be also detected in the apical membrane (arrow heads) and some vesicles (arrows) (Fig. 2D).

### Modification by SUMO is increased under stress conditions

**Figure 3:**
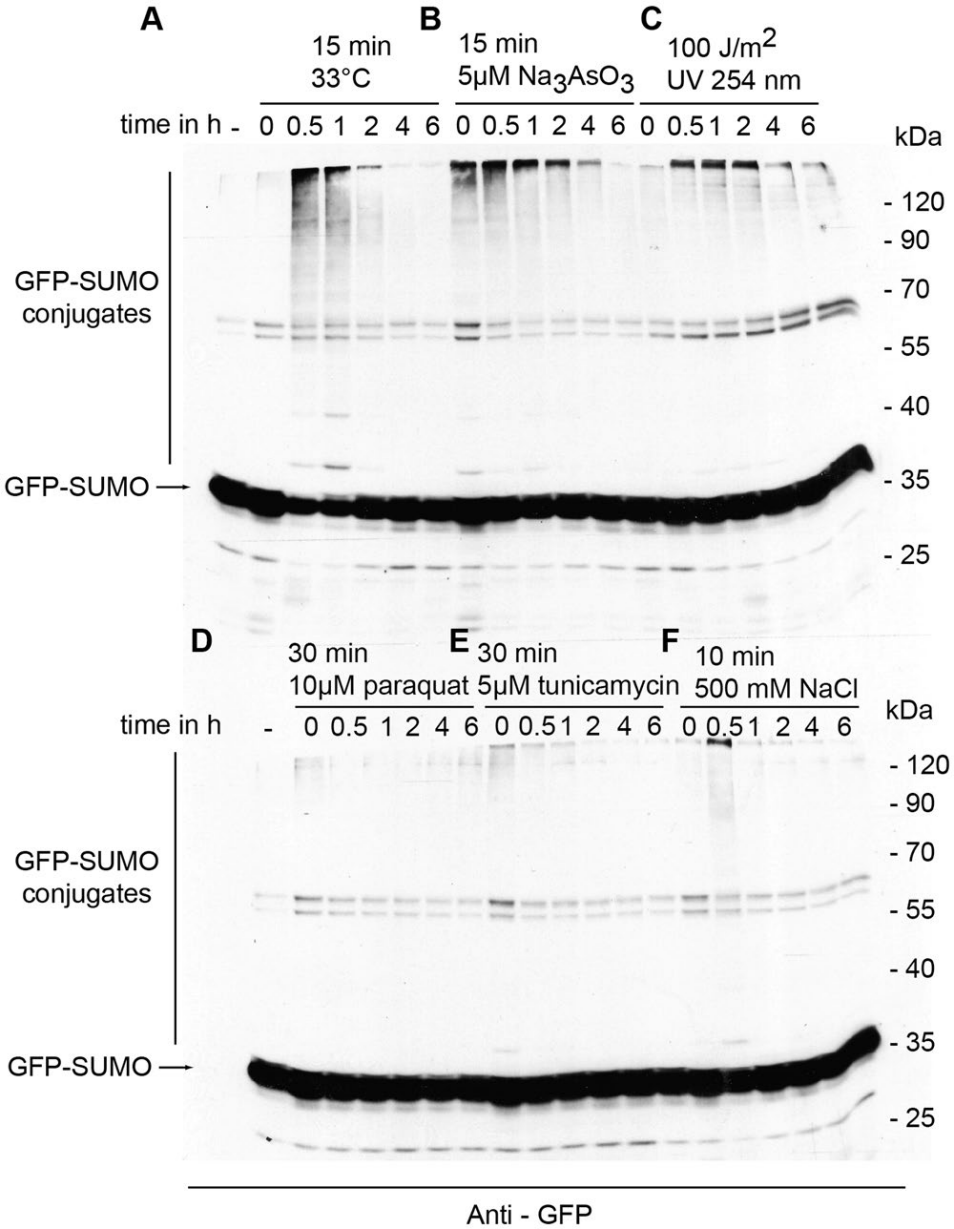
SUMO conjugation to target proteins response to stress conditions. (**A–F**) Mixed stage worms of RU86 strain were exposed to stress conditions: (**A**) heat shock, (**B**) Na_3_As03, (**C**) ultraviolet light, (**D**) paraquat, (**E**) tunicamycin and (**F**) 0.5 M NaCl. Worms were harvested at indicated time points after exposure to the stress. Control, non-treated worms indicated by **-**.Total protein lysate of worms was separated by SDS-page and immunodecorated with anti-GFP antibody.

We analyzed the levels of SUMO modification applying various stress conditions to *C. elegans*. We studied the stress response throughout time after applying short, non-lethal stress and followed it until the level of SUMOylation reverted to steady state levels (Fig. 3, Fig. S1). SUMOylation was dramatically increased in response to heat shock (Fig. 3A), arsenite induced cellular damage (Fig. 3B), and UV induced DNA damage (Fig. 3C). Short osmotic stress (10 min 500 mM NaCl) induced also transient increase in SUMOylated proteins, albeit to a lower extent (Fig. 3F). Oxidative stress induced in the mitochondria by applying paraquat did not induce global increase in SUMOylation (Fig. 3D). Endoplasmic reticulum (ER) stress induced by tunicamycin initially increased SUMOylation to a small extend, followed by decreased SUMOylation (Fig. 3E). The period of elevated SUMOylation upon transient stress condition varied depending on the stressor, from less than an hour in case of osmotic stress to over 6 hours in case of UV response. We confirmed the increase in SUMOylation in response to heat-shock in wild-type N2 worms using SUMO specific antibodies (Fig. S2).

### Identification of SUMO targets in *C. elegans*

Our stress assays in worms showed increased amount of SUMO-conjugated proteins. Thus, we reasoned that the analysis of SUMO-conjugated proteins under stress conditions may yield to yet undiscovered SUMO protein targets in worms. Using the strain RU86 expressing HIS-GFP tagged SUMO we set up the purification of proteins modified by SUMO using immobilized metal ion affinity chromatography (IMAC). Control protein purifications and identifications from the wild-type N2 worms revealed several proteins bound to the IMAC column in a non SUMO-GFP specific manner (Table S1). Indeed, *C. elegans* proteome encodes over 500 histidine rich proteins. Thus, we analyzed in parallel proteins purified from SUMO-GFP tagged transgenic worms and N2 wild-type worms (Table S2). We applied single step IMAC purification and compared the identified proteins with the wild-type control (Fig. S3A). Denaturing lysis conditions of boiling 1% SDS followed by SDS precipitation^34^ and single step purification using IMAC followed by comparison with proteins isolated from wild-type worms resulted in more specific target identification than purification in native condition, first by IMAC, followed by antibody purification. Lysis in boiling 1% SDS should abolish all protein-protein interactions and thus remove all SUMO or SUMO modified interacting proteins from the purification. Example purification is presented in Fig. S3B. SUMO conjugates were identified by mass spectrometry and data were analyzed using MaxQuant (Fig. S3A). To consider a protein as being SUMOylated, the proteins had to be identified in at least 3 independent experiments, each protein identified with at least two peptides, FDR below 1% and, not present in any of the control purifications from wild-type N2 worms or present with raw intensity greater than 10 times than in control purifications. The danger of this strategy is that some of the genuine targets that were accidently purified from control worms are excluded but we expected to identify very few falls positives.

We applied our purification and identification approach to analyze SUMO conjugates in *C. elegans* under stress conditions. We analyzed the response upon treatment of worms when global changes in SUMOylation were the strongest, namely heat shock, arsenite treatment and UV irradiation (see also Fig. 3). For each condition a non-treated control population was analyzed. To exclude proteins that non-specifically were purified, we analyzed wild-type worms under steady state and heat shock conditions. Each described condition was repeated 3 times independently. Altogether, we have analyzed 31 independent purifications from worms carrying the transgene for the tagged SUMO protein and 6 independent purifications from wild-type N2 strain (3 from non-treated and 3 from heat shock treated worms). Altogether, we have identified 874 proteins modified by SUMO in *C. elegans* in normal growth conditions and upon stress (Table S2). Unfortunately, variability of biological replicates prevented us from quantifying the differences between stress conditions in a statistically significant manner (Figs S4 and S5). Therefore, we analyzed only qualitative changes of presence/absence of an identified protein in a particular condition (Table S3). For a protein to be included in the difference list, it had to be identified in at least 2 out of 3 replicates in one condition and in none of the compared condition. Relatively low number of differentially identified proteins suggests that targets of SUMOylation in normal and stress conditions are similar but the level of modification in stress conditions is highly increased. Furthermore, proteins found to be modified in normal conditions or shortly after stress (30 min) are often not modified 120 min after stress.

**Figure 4:**
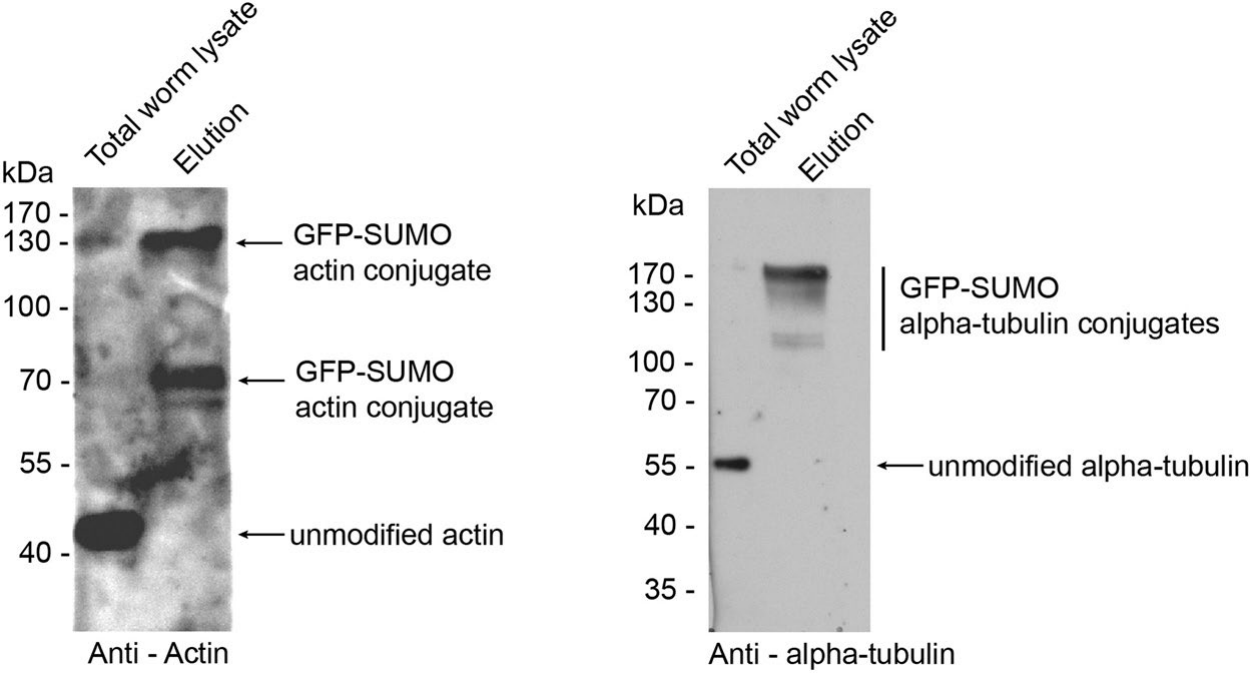
Validation of actin and alpha-tubulin as SUMO targets. Total worm lysate and elution of the purification of GFP-tagged SUMO was analysed by western blot and decorated with specific antibodies.

To validate our mass spectrometry data we used several antibodies to detect specific SUMO conjugated proteins. We confirmed SUMOylation of actin, alpha tubulin, mannosidase II, GRP94, catalase and cytochrome c1 which have been previously identified as SUMO targets in human cells (Figs 4 and S6).

Our study showed only a 25% (63 common, 810 specific this work, 185 specific for previously published targets^26^) overlap of SUMO conjugated proteins with the only present work on SUMO targets in *C. elegans*^26^. Thus, we conclude that the number of identified SUMO target proteins in worms is not saturated yet. Applying different purification strategies can yield in jet more comprehensive list of SUMO modified proteins in *C. elegans*.

### Functional analysis of SUMOylated proteins in *C. elegans*

**Figure 5:**
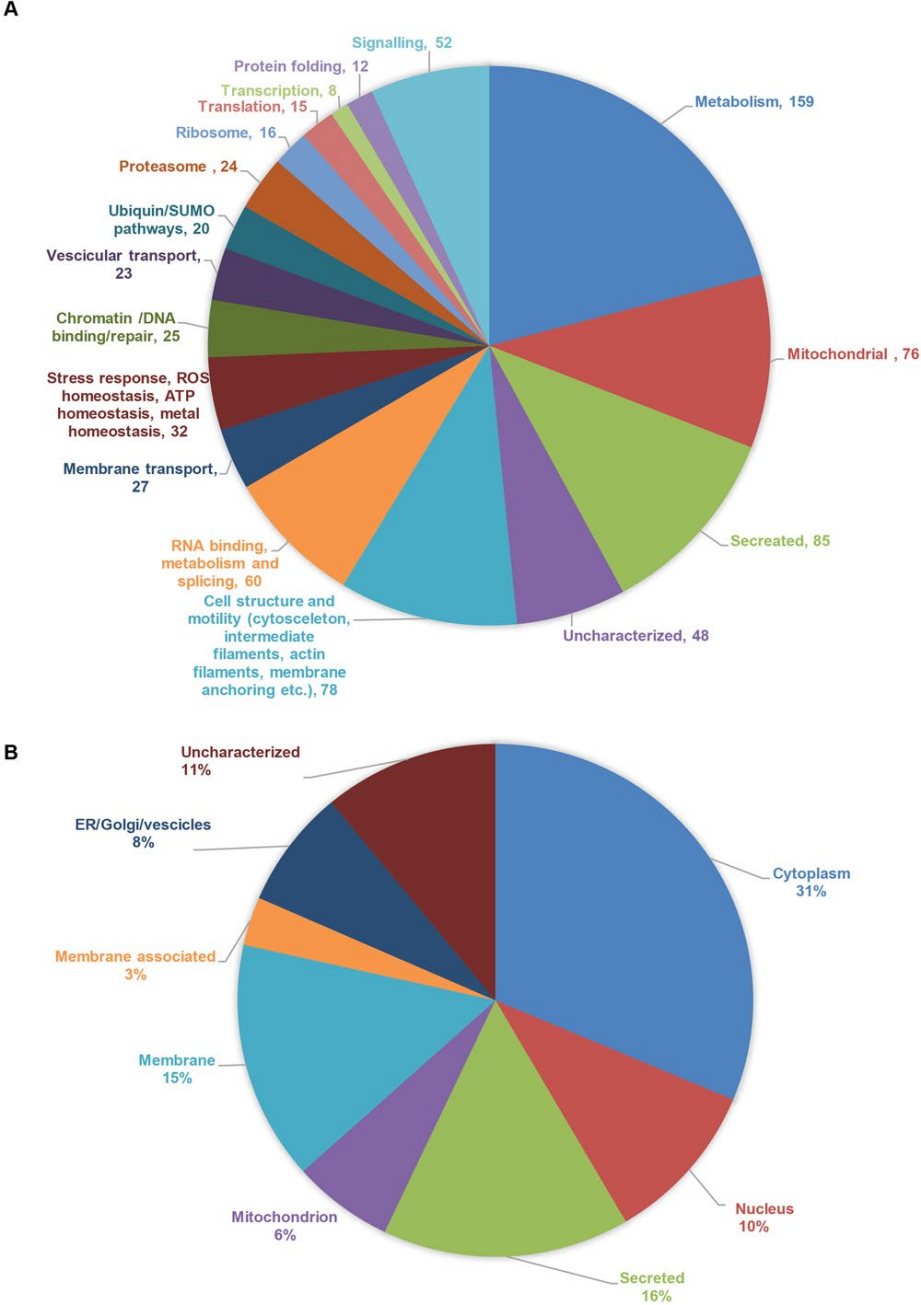
SUMO modifies targets involved in a broad range of cellular processes in the nucleus, cytosol and at membranes in *C. elegans*. (**A**) Grouping of identified SUMO targets in broad functional classes. (**B**) Grouping of identified SUMO targets based on cellular localization.

In our study we identified 874 proteins modified by SUMO in *C. elegans*. Gene ontology analysis showed that we identified proteins involved in early development and reproduction (Fig. S7C). The regulation of proteins during *C. elegans* development is in agreement with our data on the ubiquitous expression of SUMO in worms, especially high in developing embryo. Further, we identified nuclear proteins involved in genome stability, cell cycle progression, chromatin maintenance and modification, RNA splicing and ribosome biogenesis (Fig. 5A). Albeit, most of SUMOylation in the RU86 strain took place in the nucleus, the vast majority of the identified proteins were non-nuclear. Surprisingly, to a large extend these proteins are mitochondrial or extracellular (Figs 5B and S7A). None of the mitochondrial proteins identified was mitochondrially encoded though, suggesting involvement of SUMO in the biogenesis of mitochondrial proteins prior their translocation into the organelle.

Cytosolic proteins undergoing SUMOylation included proteasomal and ribosomal proteins; also proteins involved in metabolism, signaling, cell morphology and motility (cytoskeleton, microtubules, intermediate filaments and proteins involved in connecting cytoskeleton to the plasma membrane). Several membrane transport and vesicular transport proteins were also found to be modified by SUMO. Moreover, proteins involved in stress response, ROS homeostasis and proteostasis were regulated by SUMOylation (Figs 5A and S7).

**Figure 6:**
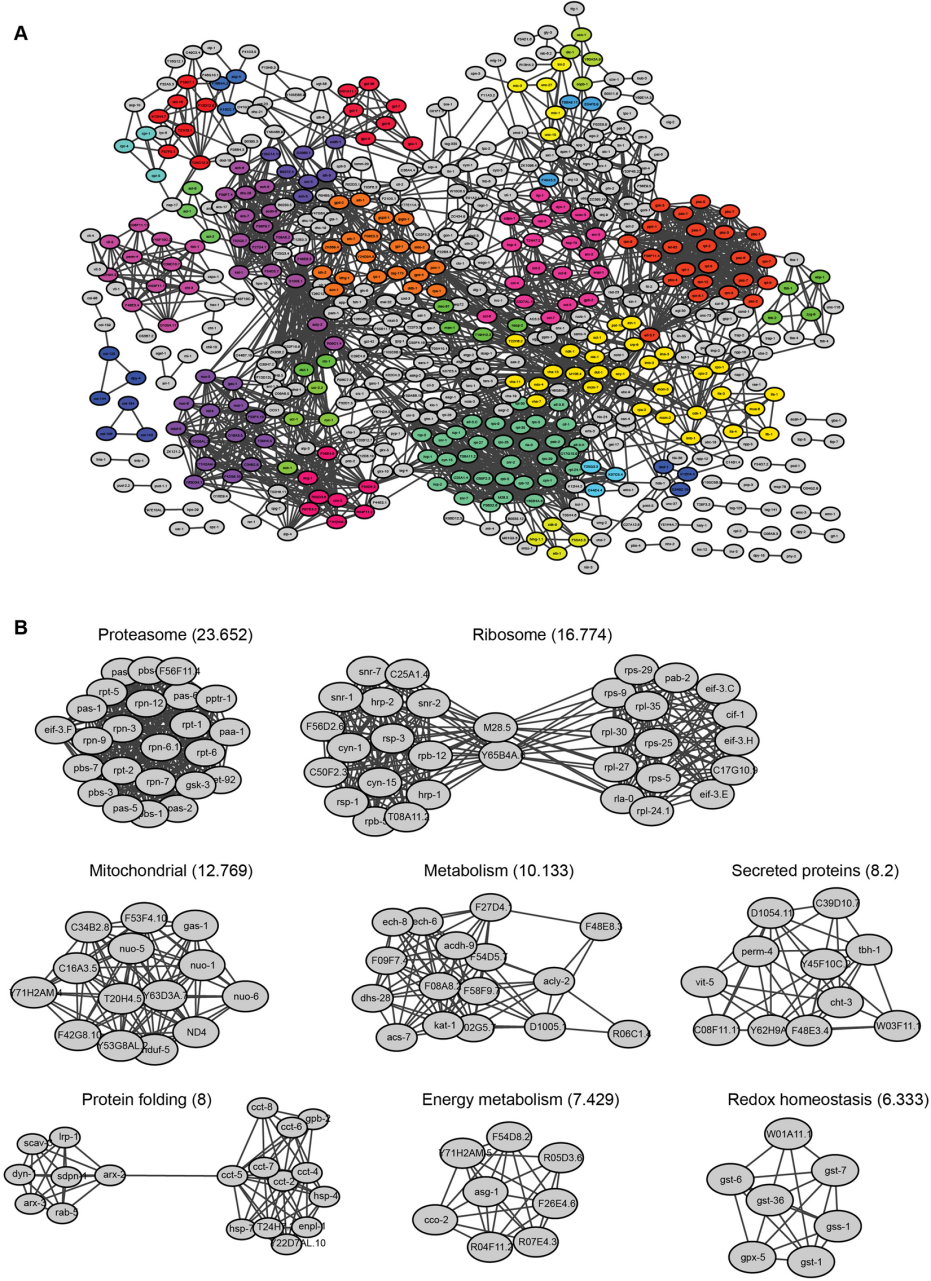
SUMO modifies highly interconnected networks of proteins in *C. elegans*. (**A**) Entire network of identified proteins analyzed by STRING database and visualized by Cytoscape. Clusters were labeled with different colors (**B**). Individual, most prominent clusters are shown. MCODE score is in parentheses.

In previous studies, proteins found to be modified by SUMO were highly interconnected^30^. We tested the interconnectedness of the identified SUMO targets in our study using the STRING database^35^. Indeed, the identified *C. elegans* proteins modified by SUMO are highly interconnected (Fig. 6A). The most prominent clusters included the proteasome, ribosome biogenesis and redox regulation (Fig. 6B). Such robust modification of proteasomal proteins was previously unidentified. Close inspection of proteasomal proteins modified by SUMO showed that most of proteins forming the core subunit as well as proteins from the base and the lid were SUMO conjugated.

In summary, we identified the largest number of SUMOylated proteins in *C. elegans* up to now including previously unnoticed classes of potential SUMO targets such as proteasomal proteins as well as mitochondrial and secreted ones.

### Conservation of SUMO target proteins

To address the question of conservation of SUMO target proteins and relevancy of research on SUMO in *C. elegans* we analyzed how evolutionary conserved are the SUMO targets in the worm in comparison to the overall protein conservation between species. We compared the list of SUMO modified proteins in the worm to the yeast^36^, *Drosophila*^37^, *Xenopus*^38^, *Arabidopsis*^39^, human^33^ and mouse^40^ proteomes using a strict definition of an ortholog requiring BLASTP e-value below 10^−10^ along at least 80% length of the proteins (Table 1). Applying these strict conditions, approximately 65% of SUMO targets in *C. elegans* have homologues in human proteome. This represents high enrichment compared to approximately 8% of homology of entire proteomes using the same conditions of comparison.

**Table 1:** Evolutionary conservation of *C. elegans* SUMO targets with other species.

Species	Proteome	Homologues in *C. elegans* proteome	Homologues in *C. elegans* SUMO targets	Enrichment p-value
*C. elegans*	26723			
*H. sapiens*	72874	6010	570	>2.2 × 10^−16^
*M. musculus*	50311	5977	580	>2.2 × 10^−16^
*X. laevis*	16378	4939	503	>2.2 × 10^−16^
*D. melanogaster*	21977	5066	529	>2.2 × 10^−16^
*A. thaliana*	31392	2993	393	>2.2 × 10^−16^
*S. cerevisiae*	6838	1886	275	>2.2 × 10^−16^

*C. elegans* proteome and SUMO targets from *C. elegans* were compared to whole proteomes and to orthologues of *C. elegans* proteins in other species. Only strict homologues with BLASTP e-value smaller than 1 × 10^−10^ and over 80% of residues in both sequences included in the BLASTP alignment, were included in the comparison.

Since *C. elegans* carry only one SUMO gene and vertebrates 4, we asked if worm SUMO targets are more conserved with human SUMO1 or SUMO2/3 targets. We found that in humans, homologues of worm SUMO targets are modified both by SUMO1 and SUMO2/3 with no statistical significant bias.

### SUMO target predication by extrapolation from other species

Our and others’^30^ analyses showed remarkable evolutionary conservation of SUMO targets throughout species. We assumed that if SUMO targets are so evolutionary conserved and the processes they are involved are also conserved, regulation of this proteins by SUMOylation might also be conserved. Thus, we attempted to predict occurrence of SUMO modification based on the experimental knowledge about the modification in any of the previously analyzed species. We applied a strict definition of an ortholog requiring BLASTP e-value below 10^−10^ along at least 80% length of the proteins. This extrapolation predicts the number of SUMOylated proteins in *C. elegans* to be approximately 4735, in humans 11115, in mouse 11777, in *A. thaliana* 5663, in *X. laevis* 6579, in *D. melanogaster* 2051 and in *S. cerevisiae* 1436 (Table S4). Our prediction suggests that at least 15–20% of the eukaryotic proteome can be modified by SUMO.

We tested our prediction by analyzing the human proteins that we have predicted to be SUMOylated but were not identified in the published experimental identifications. We have analyzed if these proteins carry any of the SUMO consensuses described by Hendriks *et al*.^30^. 88% of proteins predicted carried the consensus site compared to 94% of the experimentally identified ones. In contrast, in the entire human proteome, 65% of proteins carry the consensus site and 60.5% in the part of the proteome not shown or predicted to be SUMOylated (Table 2). The p-value of this enrichment is below 2.2 × 10^−16^.

**Table 2:** Testing of SUMOylation prediction by analysis of SUMO motifs in predicted human proteins.

	Sequences	[IV]KE	[VF]K[QTEP][ED][PKE]	KE[ED]5	[ED]K	All motifs	Containing SUMOylation motif
Identified SUMO targets	4146	1343	293	41	3862	3905	94.19%
Predicted SUMO targets	7161	1348	234	24	6219	6298	87.95%
Proteome	70956	8184	1300	148	45462	46286	65.23%
Proteome minus (identified + predicted)	59649	5493	773	83	35381	36083	60.49%

Percentage of human proteins predicted in this study but not previously experimentally identified, carrying experimentally defined SUMOylation consensus; compared to the presence of consensus in identified proteins, the entire human proteome and the proteome lacking identified and predicted SUMO targets. The p-value of the enrichment is below 2.2 × 10^−10^.

### SUMO target predication web server

To facilitate access to predictions of SUMO modifications based on evolutionary conservation of known targets, we have established a publicly available web server (sumobase.mslab-ibb.pl). Sequence alignments of known and predicted SUMOylated proteins is shown is Fig. S8B. The database contains known and predicted SUMO targets in *C. elegans*, *H. sapiens*, *M. musculus, X. laevis S. cerevisiae*, *D. melanogaster* and *A. thaliana*. Screenshots of the web page are presented in Fig. S8.

## Discussion

Every dynamic protein modification depends on the developmental stage, cell type and cell cycle stage. Analysis on the level of the entire organism enables a search throughout the entire spectrum of targets and modifications. *Caenorhabditis elegans* is an excellent model to decipher the universe of the proteins and processes regulated by SUMO. *C. elegans* enables identification of targets in all tissues and developmental stages. Recent high throughput proteomics identifications from human cells (for review see Hendriks and Vertegaal^33^) could not decipher the SUMO proteome in embryonic development and many tissues, for example neurons, germline, and liver or kidney cells.

We have analyzed SUMO modifications in *C. elegans* in all developmental stages and in various stress conditions: heat shock, DNA damage, ER stress, osmotic stress and complex inhibition of many enzymes and pathways by arsenite. Other studies have shown that SUMOylation is increased in ischemia^17,41^ and drought in plants^42^. All these stressors elicit different defense responses; nevertheless all result in increased levels of SUMOylation suggesting that SUMO is a key stress response protein on which many stress resistance mechanisms converge.

We have identified 874 proteins modified by SUMO in *C. elegans*, more than 3 times more than previously reported^26^. We compared the list of proteins identified in this study with the previously published data in *C. elegans*^26^. Surprisingly, there is little overlap between the set of proteins identified by Kaminsky *et al*. This might be due to a completely different purification strategy. We found that two step purification in native conditions, using IMAC followed by antibody purification resulted in much false positive identification of proteins that were purified from wild-type N2 worms. This is, probably due to high number of poly-His and carbohydrate binding proteins in worms as well as co-purification of SUMO interacting proteins and proteins interacting with SUMO targets. In contrast, our strategy of lysing worms in boiling 1% SDS should exclude all SUMO interacting proteins as well as proteins interacting with SUMO modified proteins.

We identified several known targets of SUMOylation for example proteins involved in cell cycle regulation, transcription, translation and cytoskeleton confirming the conservation of these processes being regulated by SUMO. We have identified several new targets of SUMO modification related to cell homeostasis and metabolism. Metabolic processes have been shown to be regulated by SUMOylation of transcription factors governing expression of metabolic enzymes^43^. In previous proteomics studies, proteins involved in metabolism and homeostasis were also identified but at much smaller proportion than in our study. Our analysis suggests also that other cellular processes are regulated by SUMOylation. Beside the ribosome, the other macromolecular complexes heavily modified by SUMO were the proteasome and proteins involved in maintenance of cell shape and motility.

In this study, we have identified several mitochondrial proteins as well as extracellular proteins. It is surprising because SUMOylation is a strictly nuclear and cytosolic process. However, recently it was shown that several inner mitochondrial proteins undergo ubiquitin mediated proteasomal degradation^44^. Furthermore, proteins can be exported from the mitochondrion for proteasomal degradation^45^. SUMO tagging is also known to indirectly target protein for proteasomal degradation^46^. Moreover, it was shown that accumulation of newly synthetized, misfolded proteins triggers SUMO conjugation response^47^. Thus, we propose that misfolded mitochondrial and secreted proteins are first SUMOylated, subsequently polyubiquitinated and degraded by the proteasome.

*C. elegans* SUMO does not contain SUMOylation consensus site. Yeast SUMO (Smt3) and human SUMO2/3 can form poly SUMO chains. Sequence analysis suggests that *C. elegans* SUMO is not more similar to SMT3 or to human SUMO2/3 than to human SUMO1 (Fig. S9). However, our functional analysis of identified SUMO targets, i.e. in response to stress suggests that worm SUMO can be functional related to SUMO2/3. The presence of high molecular weight SUMO-conjugates on the immunoblot might suggest that SUMO protein forms chains in *C. elegans* and thus, can be a signal for polyubiquitination and subsequent proteasomal degradation. *C. elegans* Y47G6A.31 protein show weak homology to *S. pombe* Rfp1 and human RNF4 proteins responsible for SUMO chain directed polyubiquitiantion. Another argument for *C. elegans* SUMO being a functional homologue of SUMO2/3 is that SUMO2/3, in contrast to SUMO1, in normal conditions is present as a pool of free protein and is being attached to target proteins upon stress^48^ what is also true in case of the worm SUMO protein. Thus, it is reasonable to assume that the only *C. elegans* SUMO performs all functions reserved for both SUMO1 and SUMO2/3 in vertebrates.

SUMOylation is a very dynamic process depending on developmental state of an organism as well as internal and external state of the cells. Precise mass spectrometry identification of SUMO modified proteins in vertebrates would require isolating and identifying SUMO conjugates from all cells and tissues in all developmental stages of a mouse, for example. Knowledge of close to complete list of SUMO targets is essential if SUMO pathway is to become a target of therapeutic interventions in humans. Thus, several groups attempted to predict potential SUMOylation based on the presence of SUMO modification consensus sites and other sequence features^49,50^. These attempts are usually hampered by the fact that 65% of proteins in the human proteome carry the SUMOylation consensus. In recent years almost 4000 human proteins have been experimentally identified to be SUMOylated. A major limitation of these identifications is that SUMO conjugates are isolated from few cell lines. Thus, our approach to identify SUMO modified proteins at the level of entire higher eukaryotic organism, at all stages of development; combined with prediction based on evolutionary conservation of targets is a useful step towards deciphering the entire SUMOylonome.

We predict that at least 15–20% of the eukaryotic proteome can be SUMOylated and suggest that SUMO functions in 3 main areas: regulation of activity of individual proteins, biogenesis of macromolecular complexes and SUMO directed proteasomal degradation.

## Methods

### *C. elegans* and bacterial strains

Worms were maintained and handled in standard conditions as described by Brenner^51^. Worms were cultured at 20 °C unless stated otherwise on plates seeded with *E. coli* HB101 bacteria. Strains used in this study: N2 (wild type) and VC186[*smo-1*(ok359)/szT1 [*lon-2*(e678)] I; +/szT1 X] (provided by the *Caenorhabditis* Genetics Center), RU86[*smo-1*(ok359); Is(p*smo-1*::*smo-1gfp*::3′UTR*smo-1*].

### Constructs and transformation

The 8-HisGFP tagged SUMO was constructed as follows: The SUMO coding sequence and 3′UTR was amplified from the worm genomic DNA with the primers 5′ ACTCCCGGCTAGCACGATGGCCGATATGC and 3′ GGACGGAGAAGGCCTTCGAATCTCGTGTC and cloned into NheI and StuI sites of the pPD117.01 vector. The 8-His tag was introduced into pPD117.01 vector (Fire kit) by PCR together with the SUMO promoter. The SUMO promoter (1 kb) was amplified from the genomic DNA with 5′-ATTTTATCACGGGCATGCTGGCCTTCCTC, 3′-CATGCTACCACACCATCACCATCACCATGCCGATGATGCAGCTCAAATTC primers and cloned into SphI and KpnI siteS of the pPD117.01 vector (Fire). The 3′ primer introduced 8His tag at the N-terminus of the GFP.

VC186 SUMO knock-out strain was transformed by biolistic transformation as previously descried^52^. Integrated transformers were selected by repeated sorting of GFP positive animals with the COPAS worm sorter (Union Biometrica) for 40 generations. Integrated strain was back-crossed 10 times with wild type N2 worms.

### Sumo conjugate purification

SUMO conjugates were purified under SDS denaturing condition as described^34,53^. Briefly, worms were grinded in liquid nitrogen and resuspended in boiling SDS lysis buffer (25 mM NaPO4 pH 7.5, 300 mM NaCl, 1%SDS) and sonicated at 20% for 2 min with Branson sonifier (Branson). Next, the lysate was spun down for 30′ at 20000 g at 4 °C. Supernatant was transferred to new tubes and cooled on ice for at least 30′ to precipitate the SDS. The lysate was cleared of precipitated SDS by centrifugation at 10000 g for 30′ at 0 °C. The cleared lysate was filtered through 0.22 μ filter and applied to IMAC column (GE Healthcare) equilibrated with the buffer (25 mM NaPO4 pH 7.5, 300 mM NaCl, 20 mM imidazole. 0.1% sarcosyl). The column was washed with at least 20 volumes of the wash buffer (25 mM NaPO4 pH 7.5, 300 mM NaCl, 50 mM imidazole. 0.1% sarcosyl). Proteins were eluted with 2 column volumes of the elution buffer (25 mM NaPO4 pH 7.5, 300 mM NaCl, 300 mM imidazole. 0.1% sarcosyl).

### In-solution digestion by FASP

Isolated proteins were prepared for mass spectrometry analysis by FASP (Filter Assisted Sample Preparation)^54^. Briefly, 50 μg of each sample was reduced with 50 mM DTT at 55 °C for 30 min, diluted 1:8 in 8 M urea and applied to Vivacon 500 ultrafiltration unit MWCO 30000 (Sartorius). Samples were washed twice with 8 M urea, free cysteins were blocked with 50 μM iodoacetamide in 8 M urea and the filters were washed twice with 8 M urea and twice with 50 mM NH_4_HCO_3_. Next, the samples were digested in a wet chamber, O/N with trypsin (Promega) in 50 mM NH_4_HCO_3_, protein: trypsin ratio 100:1.

### Mass spectrometry

Peptide mixtures were applied to RP-18 precolumns (nanoACQUITY Symmetry® C18—Waters) using water containing 0.1% trifluoroacetic acid as mobile phase and then transferred to nano-HPLC RP-18 columns (nanoACQUITY BEH C18—Waters) using an acetonitrile gradient (5–35%) for 180 min in the presence of 0.05% formic acid with a flow rate of 250 nl min^−1^. The column outlet was directly coupled to the ion source of Q Exactive™ Hybrid Quadrupole-Orbitrap Mass Spectrometer (Thermo Electron Corp). The mass spectrometer was operated in positive ion mode with a selected mass range of 300–2000 mass/charge (m/z).

### Data processing analysis

Raw files were processed, including peak list generation, using the MaxQuant (v1.5.7.4) computational proteomics platform and default parameters were used. The fragmentation spectra were searched using Andromeda search engine integrated into the MaxQuant platform against a custom, non-redundant database of *C. elegans* proteome that included the sequence of the SUMO-GFP protein. The error ranges for the first and main searches were 20 ppm and 6 ppm, respectively, with 2 missed cleavages. Carbamidomethylation of cysteines was set as a fixed modification, and oxidation and protein N-terminal acetylation were selected as variable modifications for database searching. The minimum peptide length was set at 6 aa. Both peptide and protein identifications were filtered at a 1% false discovery rate. Enzyme specificity was set to trypsin.

The mass spectrometry proteomics data have been deposited to the ProteomeXchange Consortium via the PRIDE^55^ partner repository with the dataset identifier PXD006644.

### Stress assays in *C. elegans*

Heat shock was induced by 15′ incubation in at 33 °C in a water bath. UV stress was induced by irradiation of 100 J/m^2^ UV 254 nm in a Stratalinker (Stratagene). Arsenite treatment 30′ 5 μM sodium arsenite (Sigma) in M9. Osmotic stress was induced by incubation in 500 mM NaCl in M9 for 10′. ER stress was introduced by 30′ incubation in 5 μM tunicamycin in M9. Oxidative stress was introduced by 30 min incubation in 10 μM paraquat in M9.

### Microscopy

Worms were anesthetized in 20 mM sodium azide in M9 and immobilized on 2% agarose pads. Fluorescent images were taken with Zeiss Observer D1 microscope 40 × 0.6 lens, excitation 480 nm LED light source, Confocal images were takes with Olympus FV1000 microscope (Olympus) 60 × 1.4 oil immersion lens. Excititation 440 nm. The images were processed with ImageJ v 1.51n.

### Immunoblot analysis

Worms were collected at different time points in M9 buffer, spun down and equal volume 2x SDS lysis buffer (50 mM NaPO4 pH 7.5, 600 mM NaCl, 2%SDS) was added. Worms were frozen in liquid nitrogen, thawed and sonicated at 10% for 2 min with Branson sonifier (Branson). The lysates were spun down. The protein content was measured with BCA kit (Thermo Scientific). 20 μg of protein lysate was loaded on each lane and separated by SDS-PAGE followed by electroblotting on PVDF membrane. The membrane was blocked with 5% non-fat milk and incubated with anti-GFP antibodies (Roche) o/n followed by horse radish conjugated secondary antibody. Rabbit antibody against *C. elegans* SUMO was generated against the N-terminal peptide MADDAAQAGDNAEYIKIK (Eurogentec). Antibodies used to confirm the SUMO modification included: anti actin (MAB1501 from Chemicon), anti alpha tubulin clone DM1A (Sigma T9026), anti mannosidase II (AbDSerotec AHP674), anti cytochrome C1 (custom made), anti catalase (SantaCruz sc-365738) anti GRP94 (SantaCruz sc-11402). The secondary antibody was detected with ECL system.

### Bioinformatics analysis

Network analysis was performed using STRING database^56^, minimal interactions score was set to “high confidence”. STRING generated networks were further analyzed by Cytoscape^57^.

Proteins were considered as orthologues when the BLASTP E-value was smaller than 1 × 10^−10^ and over 80% of residues in both sequences included in the BLASTP alignment^58^. Enrichment analysis p-value was calculated using Pearson’s Chi-squared test with Yates’ continuity correction. The multiple sequence alignment was performed by MUSCLE software^59^.

Evolutionary analyses were conducted in MEGA7^60^. The evolutionary history was inferred by using the Maximum Likelihood method based on the Whelan And Goldman model.

## Electronic supplementary material

Supplementary Information

S1

S2

S3

S4
