# Supplementary material for: Comprehensive list of SUMO targets in Caenorhabditis elegans and its implication for evolutionary conservation of SUMO signaling
Source: Sci Rep. 2018 Jan 18;8:1139. doi: 10.1038/s41598-018-19424-9 (PMC5773548; doi:10.1038/s41598-018-19424-9)
Supplement: Supplementary file 1 — Supplementary Information [file 41598_2018_19424_MOESM1_ESM.pdf]

Comprehensive list of SUMO targets in *Caenorhabditis elegans* and its' implication for evolutionary conservation of SUMO signaling.

Krzysztof Drabikowski, Jacqueline Ferralli, Michal Kistowski, Jacek Oledzki, Michal Dadlez and Ruth Chiquet-Ehrismann.

Supplementary Figure legends

Figure S1.Changes in SUMO-conjugation upon stress conditions. Coomassie staining corresponding to Fig. 3 serves as loading control.

Figure S2. Endogenous SUMO conjugated to targets changes upon heat stress. Mixed population of wild-type *C. elegans* were heat shocked for 15 min at 33°C. A portion of non-heat shocked worms of the same mixed population serves as baseline for SUMO-conjugated targets (-). Heat-shocked worms were allowed to recover at 20°C. Samples during recovery time were collected at indicated time-points. Total protein lysate was separated by SDS-PAGE and immunodecorated with anti-SUMO antibody (left). Amido black staining serves as loading control (right).

Figure S3. Purification strategy. (**A**) Schema of purification steps from RU86 strain and wild-type worms. (**B**) Purification of GFP-SUMO (see also Methods „SUMO conjugate purification") and analysis by western blot using anti-GFP antibody. SUMO-GFP and wild-type worms were processed in parallel.

Figure S4. Comparison of biological replicates upon arsenite and UV treatment. Analysis of correlation coeffiencency between biological replicates. Correlation coeffiencency is given for each comparison in the upper left corner of each graph.

Figure S5. Comparison of biological replicates and overlap of identified proteins. (**A**) Analysis of correlation coeffiencency between biological replicates upon heat shock. Correlation coeffiencency is given for each comparison in the upper left

corner of each graph. (**B**) Venn diagrams showing overlap in identified sumoylated proteins among the biological replicates within each experimental condition.

Figure S6. Validation of SUMO targets. Total worm lysate and/ or elution of the purification of GFP-tagged SUMO was analysed by western blot and decorated with specific antibodies.

Figure S7. Gene ontology (GO) analysis. (**A-D**) Terms were considered as enriched when corrected p-Value (Benjamini-Hochberg) was <0.05. Numbers on the bar charts indicate the number of proteins within each enriched term. (**A-C**) Ontologizer software was used to analyse GO-term enrichment within all purified SUMO-conjugated protein (see also Table S2) and compared to entire proteome of *C. elegans*. (**D**) KEGG-pathway analysis using DAVID functional annotation tool.

Figure S8. Screenshots of the webpage. (**A**) Main page of the sumobase.mslab-ibb.pl web server. (**B**) Example of output from a protein entry.

Figure S9. Evolutionary history of SUMO proteins. (**A**) Alignment of *C. elegans* SUMO, *S. cerevisiae* SMT3, *H. sapiens* SUMO1 and SUMO2. (**B**)**.** Bootstrap consensus tree inferred from 2000 replicates. Branches corresponding to partitions reproduced in less than 50% bootstrap replicates are collapsed. The percentage of replicate trees in which the associated taxa clustered together in the bootstrap test (2000 replicates) are shown next to the branches.

Supplementary Table legends

Table S1. Proteins identified in N2 worms after purification. List of proteins identified in control purifications from wild-type N2 worms. Each protein identified with at least two peptides, FDR below 1%.

Table S2. SUMO-conjugated proteins identified in normal and stress conditions. List of SUMO-conjugated proteins identified in this study. Proteins were considered as SUMOylated if they were identified in at least 3 independent experiments, each protein identified with at least two peptides, FDR below 1% and not present in any of the control purifications from wild-type N2 worms or present with raw intensity greater than 10 times than in control purifications.

Table S3: SUMO-conjugated proteins differentially identified in stress condition. List of differential SUMO-conjugated proteins identified in this study. For a protein to be included in the differential list, it had to be identified in at least 2 out of 3 replicates in one condition and in none of the compared condition.

Table S4: Identified and predicted SUMO conjugates present in the SUMObase database. Lists of identified and predicted SUMO targets in *Caenorhabditis elegans, Homo sapiens, Mus musculus, Xenopus laevis, Drosophila melanogaster, Arabidopsis thaliana and Saccaromyces cerevisiae.*

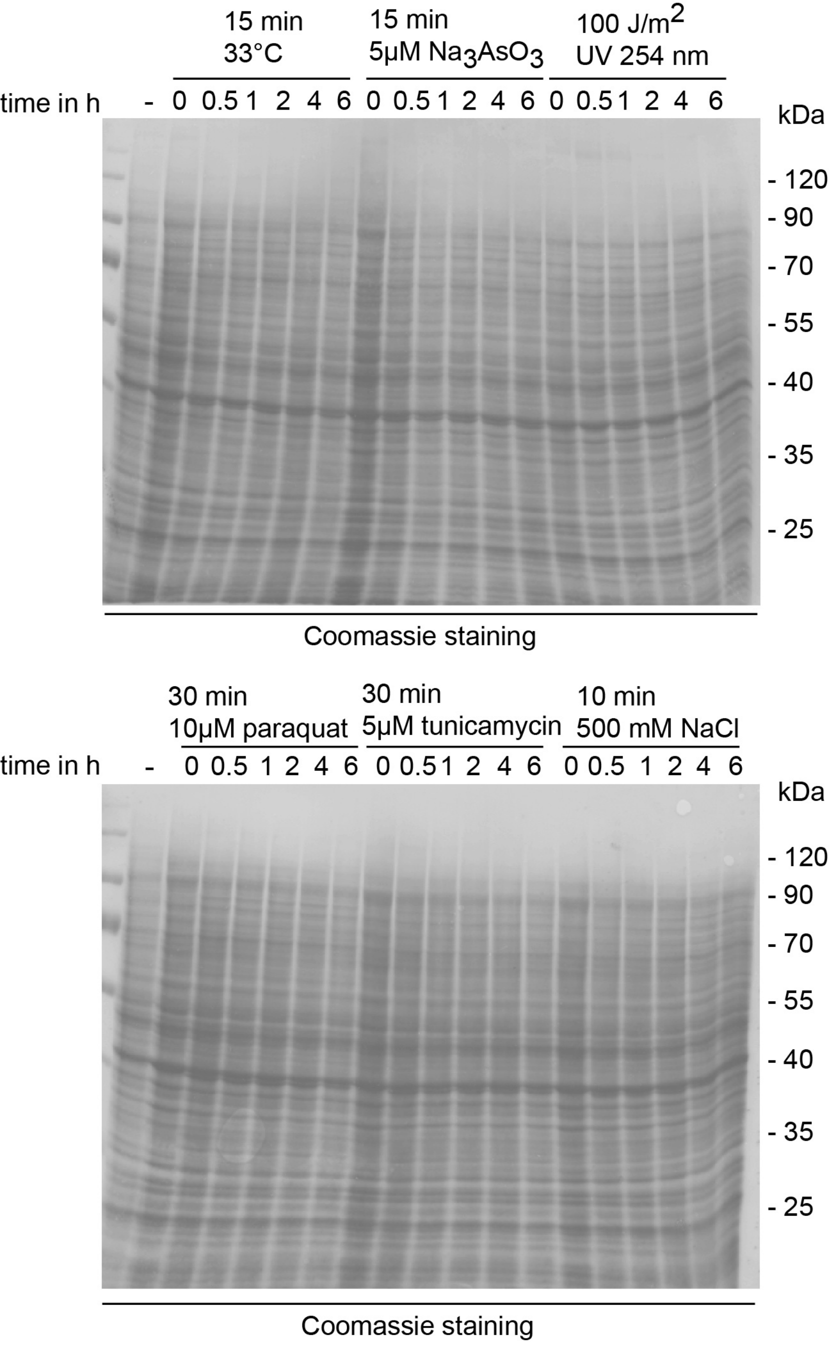
15 min
33°C
15 min
5μM Na3AsO3
100 J/m2
UV 254 nm
time in h
- 0 0.5 1 2 4 6 0 0.5 1 2 4 6 0 0.5 1 2 4 6
kDa
- 120
- 90
- 70
- 55
- 40
- 35
- 25
Coomassie staining
30 min
10μM paraquat
30 min
5μM tunicamycin
10 min
500 mM NaCl
time in h
- 0 0.5 1 2 4 6 0 0.5 1 2 4 6 0 0.5 1 2 4 6
kDa
- 120
- 90
- 70
- 55
- 40
- 35
- 25
Coomassie staining

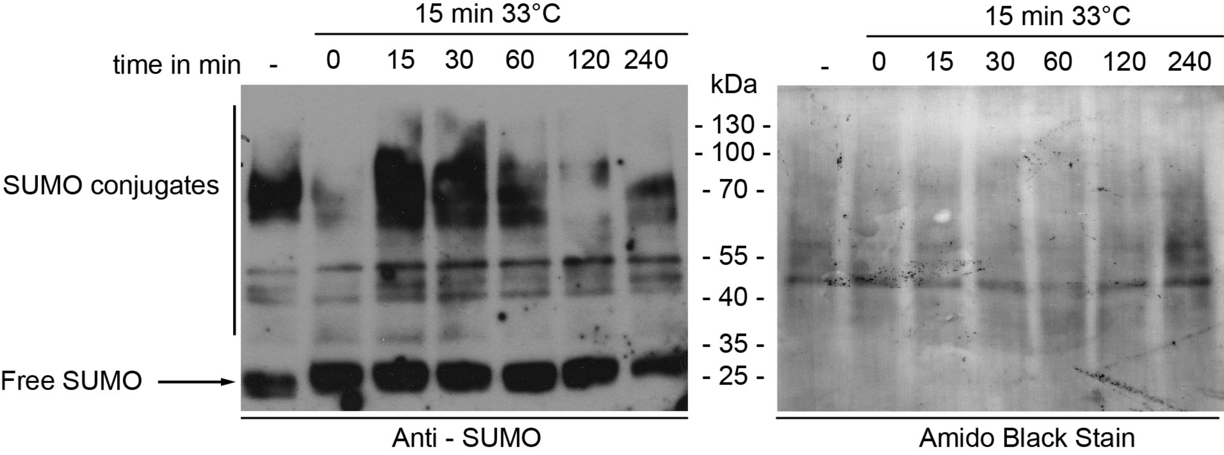

15 min 33°C
time in min
-
0
15
30
60
120
240
kDa
- 130 -
- 100 -
- 70 -
- 55 -
- 40 -
- 35 -
- 25 -
SUMO conjugates
Free SUMO
Anti - SUMO
15 min 33°C
-
0
15
30
60
120
240
Amido Black Stain

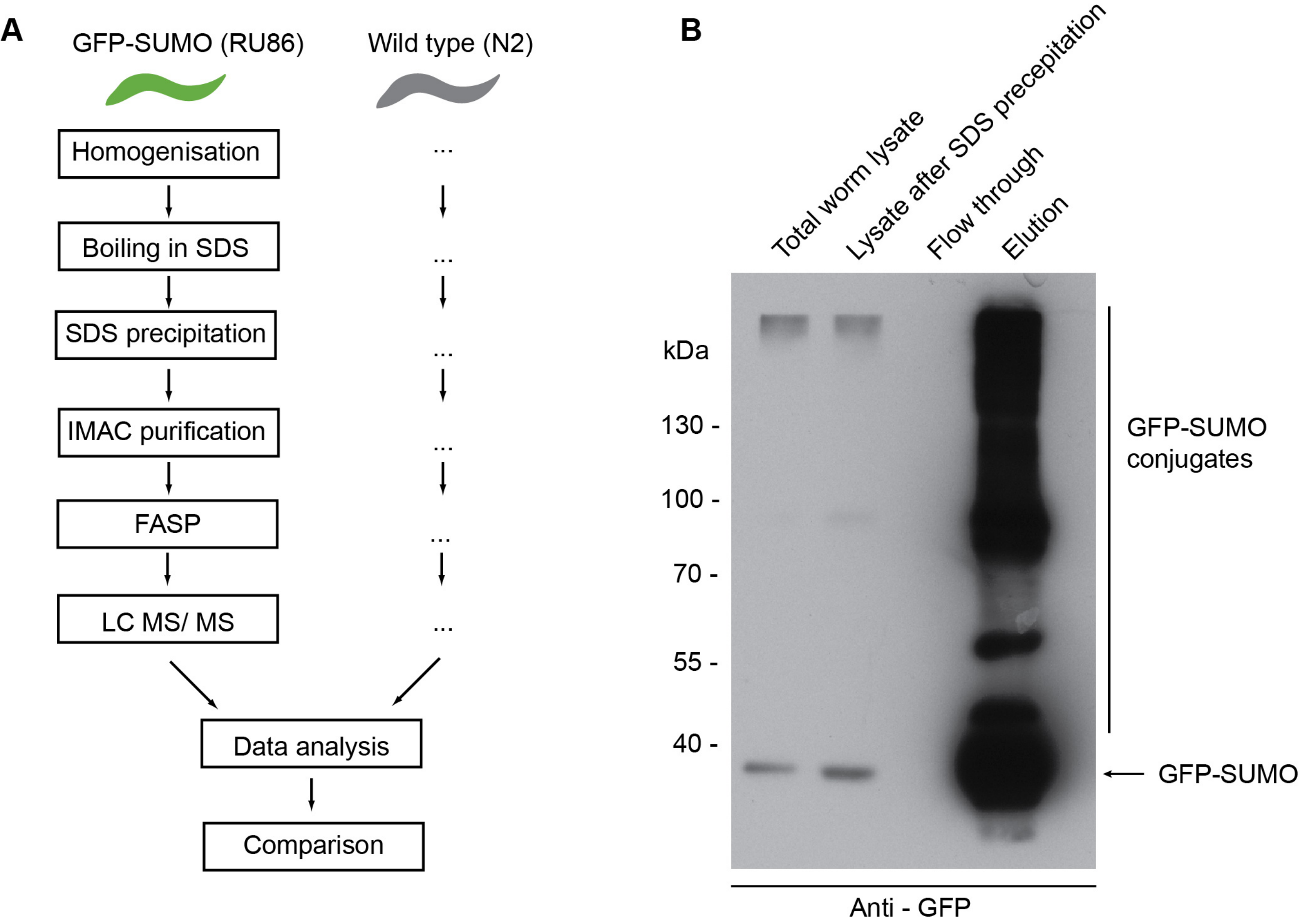
A
GFP-SUMO (RU86)
Wild type (N2)
Homogenisation
Boiling in SDS
SDS precipitation
IMAC purification
FASP
LC MS/ MS
Data analysis
Comparison
B
Total worm lysate
Lysate after SDS precepitation
Flow through
Elution
kDa
130 -
100 -
70 -
55 -
40 -
GFP-SUMO conjugates
GFP-SUMO
Anti - GFP

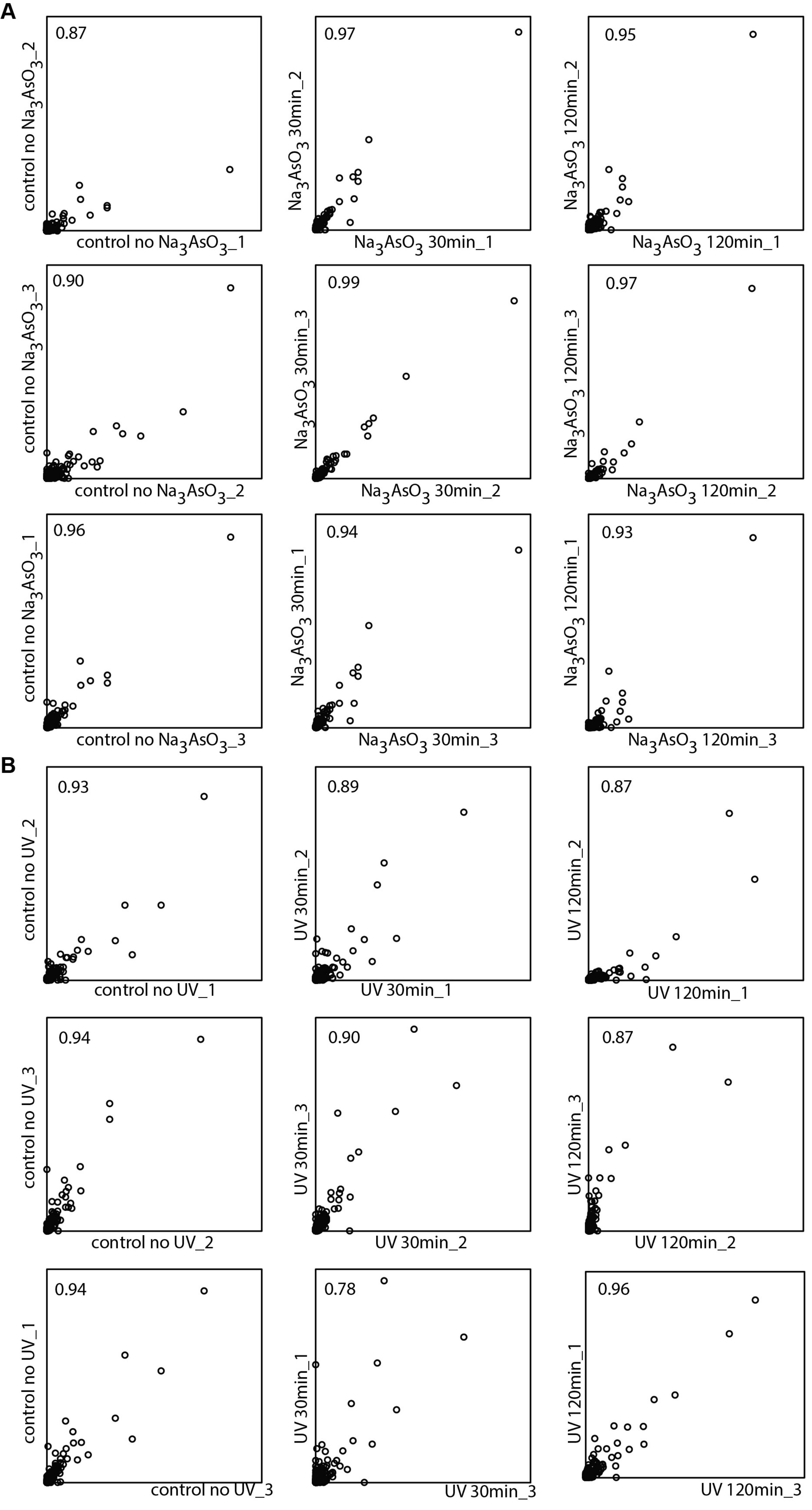
A
0.87
control no Na3AsO3_2
control no Na3AsO3_1
0.97
Na3AsO3 30min_2
Na3AsO3 30min_1
0.95
Na3AsO3 120min_2
Na3AsO3 120min_1
0.90
control no Na3AsO3_3
control no Na3AsO3_2
0.99
Na3AsO3 30min_3
Na3AsO3 30min_2
0.97
Na3AsO3 120min_3
Na3AsO3 120min_2
0.96
control no Na3AsO3_1
control no Na3AsO3_3
0.94
Na3AsO3 30min_1
Na3AsO3 30min_3
0.93
Na3AsO3 120min_1
Na3AsO3 120min_3
B
0.93
control no UV_2
control no UV_1
0.89
UV 30min_2
UV 30min_1
0.87
UV 120min_2
UV 120min_1
0.94
control no UV_3
control no UV_2
0.90
UV 30min_3
UV 30min_2
0.87
UV 120min_3
UV 120min_2
0.94
control no UV_1
control no UV_3
0.78
UV 30min_1
UV 30min_3
0.96
UV 120min_1
UV 120min_3

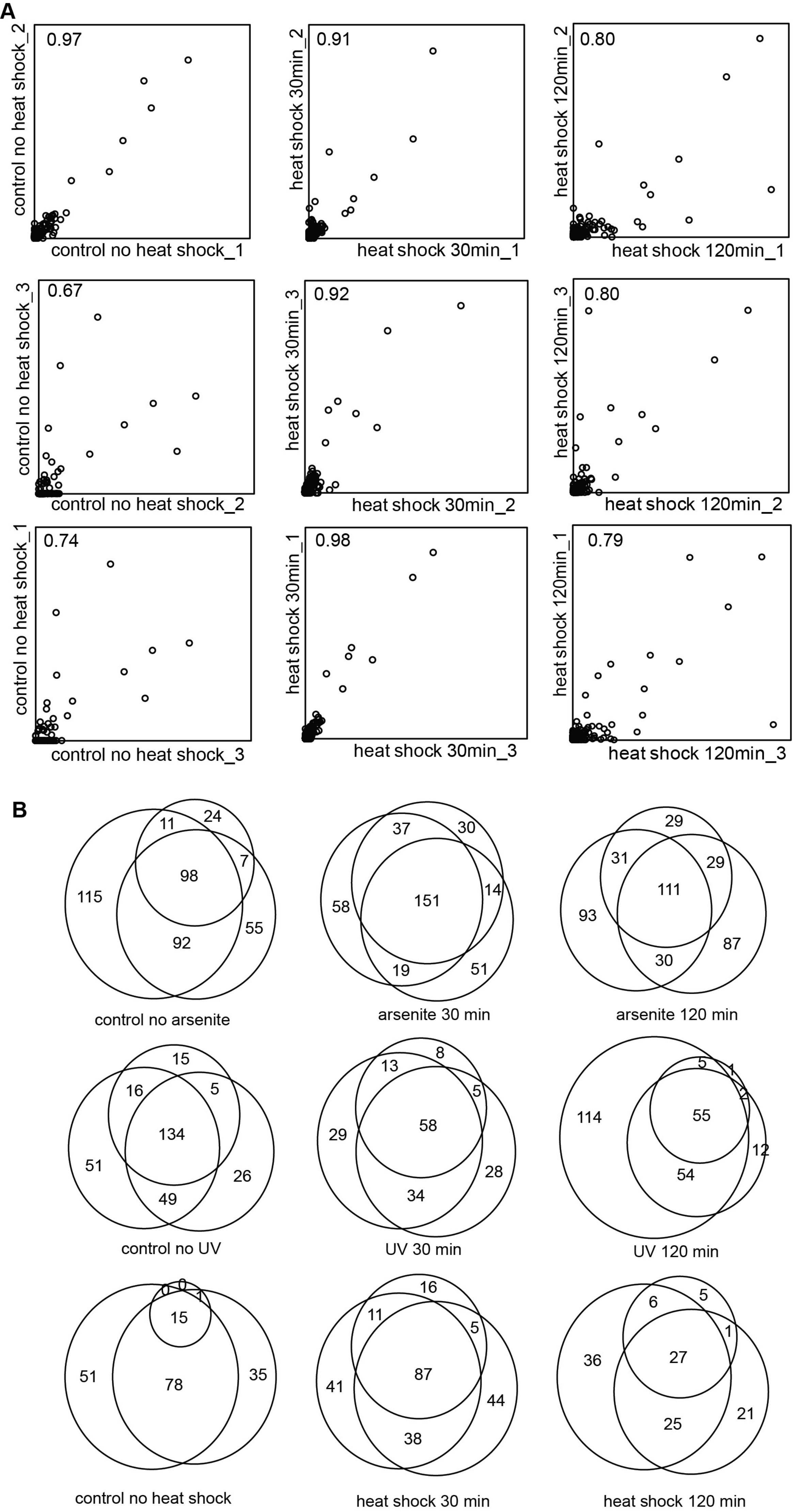

A
0.97
control no heat shock_2
control no heat shock_1
0.91
heat shock 30min_2
heat shock 30min_1
0.80
heat shock 120min_2
heat shock 120min_1
0.67
control no heat shock_3
control no heat shock_2
0.92
heat shock 30min_3
heat shock 30min_2
0.80
heat shock 120min_3
heat shock 120min_2
0.74
control no heat shock_1
control no heat shock_3
0.98
heat shock 30min_1
heat shock 30min_3
0.79
heat shock 120min_1
heat shock 120min_3
B
11
24
7
98
115
92
55
control no arsenite
37
30
58
151
14
19
51
arsenite 30 min
29
31
29
111
93
87
30
arsenite 120 min
15
16
5
134
51
26
49
control no UV
8
13
5
58
29
28
34
UV 30 min
5
1
2
55
114
12
54
UV 120 min
0
0
1
15
51
78
35
control no heat shock
16
11
5
87
41
44
38
heat shock 30 min
6
5
1
27
36
25
21
heat shock 120 min

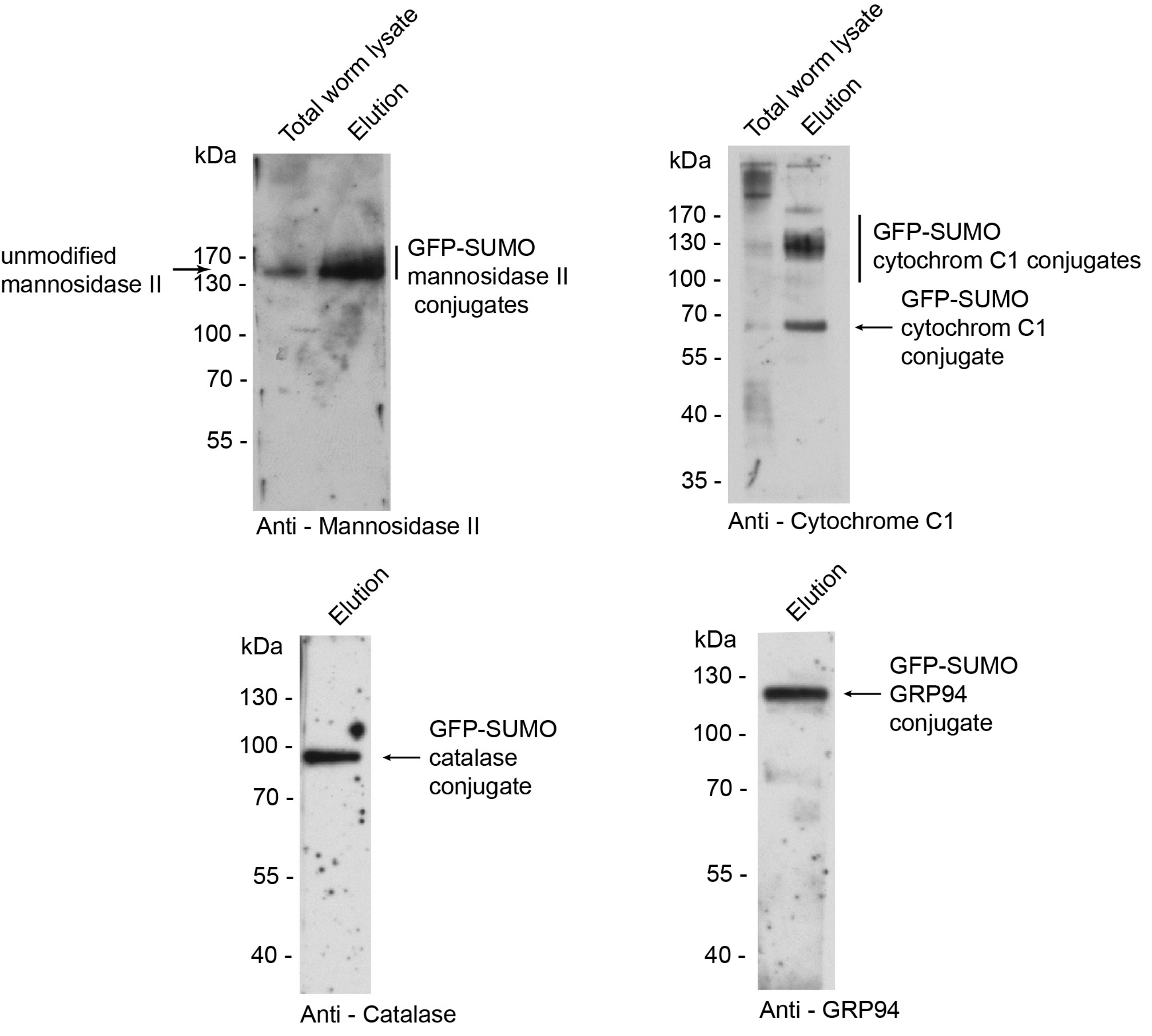
Total worm lysate
Elution
kDa
170 -
130 -
100 -
70 -
55 -
unmodified mannosidase II
GFP-SUMO mannosidase II conjugates
Anti - Mannosidase II
Total worm lysate
Elution
kDa
170 -
130 -
100 -
70 -
55 -
40 -
35 -
GFP-SUMO cytochrom C1 conjugates
GFP-SUMO cytochrom C1 conjugate
Anti - Cytochrome C1
Elution
kDa
130 -
100 -
70 -
55 -
40 -
GFP-SUMO catalase conjugate
Anti - Catalase
Elution
kDa
130 -
100 -
70 -
55 -
40 -
GFP-SUMO GRP94 conjugate
Anti - GRP94

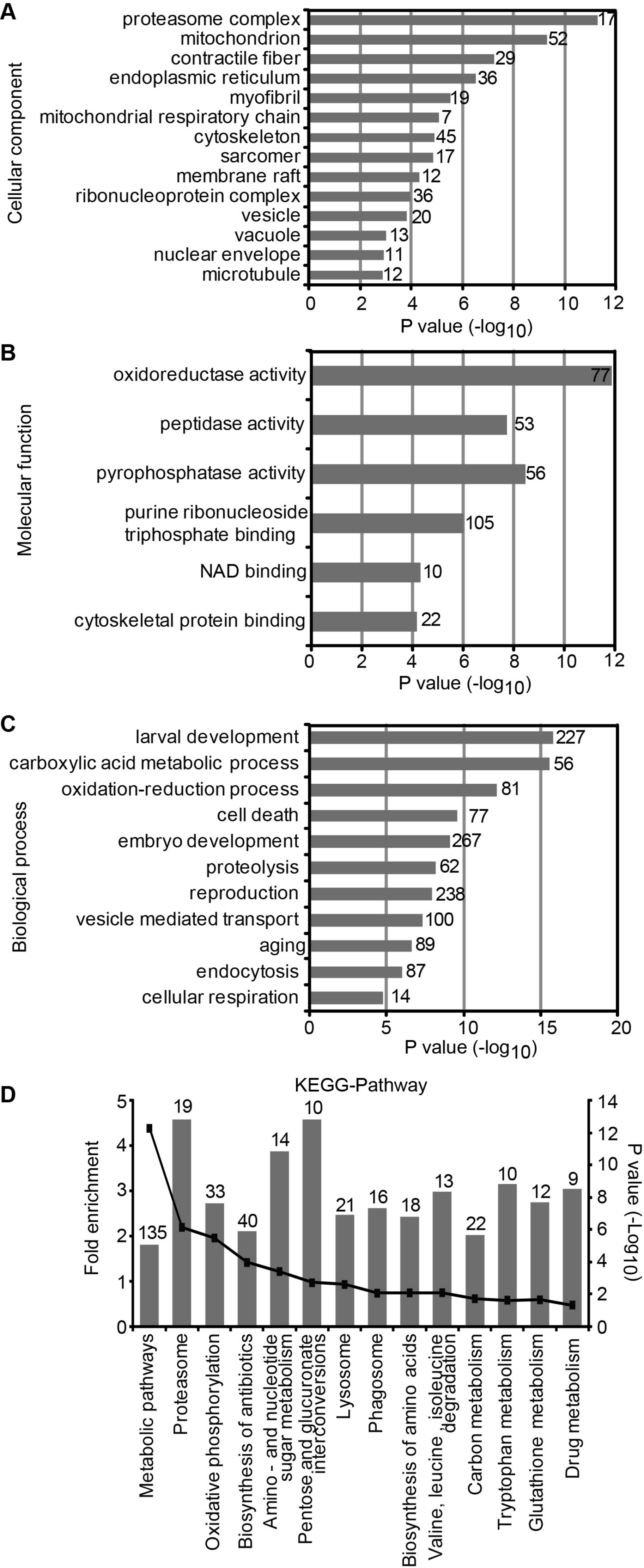

A
Cellular component
proteasome complex
mitochondrion
contractile fiber
endoplasmic reticulum
myofibril
mitochondrial respiratory chain
cytoskeleton
sarcomer
membrane raft
ribonucleoprotein complex
vesicle
vacuole
nuclear envelope
microtubule
17
52
29
36
19
7
45
17
12
36
20
13
11
12
0 2 4 6 8 10 12
P value (-log10)
B
Molecular function
oxidoreductase activity
peptidase activity
pyrophosphatase activity
purine ribonucleoside triphosphate binding
NAD binding
cytoskeletal protein binding
77
53
56
105
10
22
0 2 4 6 8 10 12
P value (-log10)
C
Biological process
larval development
carboxylic acid metabolic process
oxidation-reduction process
cell death
embryo development
proteolysis
reproduction
vesicle mediated transport
aging
endocytosis
cellular respiration
227
56
81
77
267
62
238
100
89
87
14
0 5 10 15 20
P value (-log10)
D
KEGG-Pathway
Fold enrichment
P value (-Log10)
0 1 2 3 4 5
0 2 4 6 8 10 12 14
135
19
33
40
14
10
21
16
18
13
22
10
12
9
Metabolic pathways
Proteasome
Oxidative phosphorylation
Biosynthesis of antibiotics
Amino - and nucleotide sugar metabolism
Pentose and glucuronate interconversions
Lysosome
Phagosome
Biosynthesis of amino acids
Valine, leucine, isoleucine degradation
Carbon metabolism
Tryptophan metabolism
Glutathione metabolism
Drug metabolism

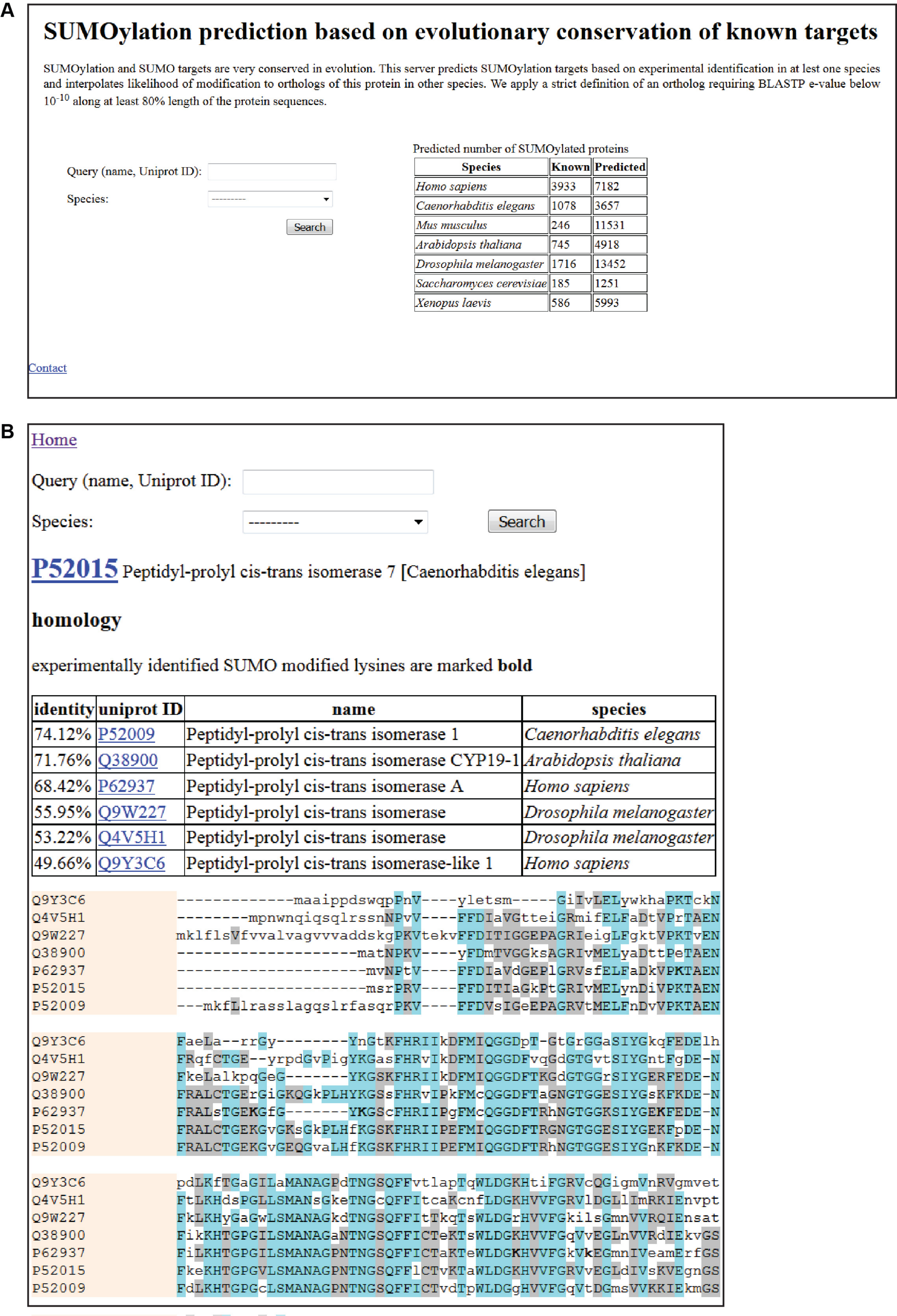

A

SUMOylation prediction based on evolutionary conservation of known targets

SUMOylation and SUMO targets are very conserved in evolution. This server predicts SUMOylation targets based on experimental identification in at lest one species and interpolates likelihood of modification to orthologs of this protein in other species. We apply a strict definition of an ortholog requiring BLASTP e-value below 10^{-10} along at least 80% length of the protein sequences.

Query (name, Uniprot ID):

Species: ---------

Search

Predicted number of SUMOylated proteins

Species	Known	Predicted
Homo sapiens	3933	7182
Caenorhabditis elegans	1078	3657
Mus musculus	246	11531
Arabidopsis thaliana	745	4918
Drosophila melanogaster	1716	13452
Saccharomyces cerevisiae	185	1251
Xenopus laevis	586	5993

Contact

B

Home

Query (name, Uniprot ID):

Species: ----------

Search

P52015 Peptidyl-prolyl cis-trans isomerase 7 [Caenorhabditis elegans]

homology

experimentally identified SUMO modified lysines are marked **bold**

identity	uniprot ID	name	species
74.12%	P52009	Peptidyl-prolyl cis-trans isomerase 1	*Caenorhabditis elegans*
71.76%	Q38900	Peptidyl-prolyl cis-trans isomerase CYP19-1	*Arabidopsis thaliana*
68.42%	P62937	Peptidyl-prolyl cis-trans isomerase A	*Homo sapiens*
55.95%	Q9W227	Peptidyl-prolyl cis-trans isomerase	*Drosophila melanogaster*
53.22%	Q4V5H1	Peptidyl-prolyl cis-trans isomerase	*Drosophila melanogaster*
49.66%	Q9Y3C6	Peptidyl-prolyl cis-trans isomerase-like 1	*Homo sapiens*

```
Q9Y3C6    -------------maaippdswqpPnV----yletsm-----GiIvLELywkhaPKTckN
Q4V5H1    --------mpnwnqiqsqlrssnNPvV----FFDIaVGtteiGRmifELFaDtVPrTAEN
Q9W227    mklflsVfvvalvagvvvaddskgPKVtekvFFDITIGGEPAGRIeigLFgktVPKTvEN
Q38900    --------------------matNPKV----yFDmTVGGksAGRIvMELyaDttPeTAEN
P62937    ---------------------mvNPtV----FFDIaVdGEPlGRVsfELFaDkVPKTAEN
P52015    ---------------------msrPRV----FFDITIaGkPtGRIvMELynDiVPKTAEN
P52009    ---mkfLlrasslagqslrfasqrPKV----FFDVsIGeEPAGRVtMELFnDvVPKTAEN

Q9Y3C6    FaeLa--rrGy--------YnGtKFHRIIkDFMIQGGDpT-GtGrGGaSIYGkqFEDElh
Q4V5H1    FRqfCTGE--yrpdGvPigYKGasFHRvIkDFMIQGGDFvqGdGTGvtSIYGntFgDE-N
Q9W227    FkeLalkpqGeG-------YKGSKFHRIIkDFMIQGGDFTKGdGTGGrSIYGERFEDE-N
Q38900    FRALCTGErGiGKQGkPLHYKGSsFHRvIPkFMcQGGDFTaGNGTGGESIYGsKFKDE-N
P62937    FRALsTGEKGfG-------YKGScFHRIIPgFMcQGGDFTRhNGTGGKSIYGEKFEDE-N
P52015    FRALCTGEKGvGKsGkPLHfKGSKFHRIIPEFMIQGGDFTRGNGTGGESIYGEKFpDE-N
P52009    FRALCTGEKGvGEQGvaLHfKGSKFHRIIPEFMIQGGDFTRhNGTGGESIYGnKFKDE-N

Q9Y3C6    pdLKfTGaGILaMANAGPdTNGSQFFvtlapTqWLDGKHtiFGRVcQGigmVnRVgmvet
Q4V5H1    FtLKHdsPGLLSMANsGkeTNGcQFFItcaKcnfLDGKHVVFGRVlDGLlImRKIEnvpt
Q9W227    FkLKHyGaGwLSMANAGkdTNGSQFFItTkqTsWLDGrHVVFGkilsGmnVVRQIEnsat
Q38900    FikKHTGPGILSMANAGaNTNGSQFFICTeKTsWLDGKHVVFGqVvEGLnVVRdIEkvGS
P62937    FiLKHTGPGILSMANAGPNTNGSQFFICTaKTeWLDGKHVVFGkVkEGmnIVeamErfGS
P52015    FkeKHTGPGVLSMANAGPNTNGSQFFlCTvKTaWLDGKHVVFGRVvEGLdIVsKVEgnGS
P52009    FdLKHTGPGcLSMANAGPNTNGSQFFICTvdTpWLDGgHVVFGqVtDGmsVVKKIEkmGS
```

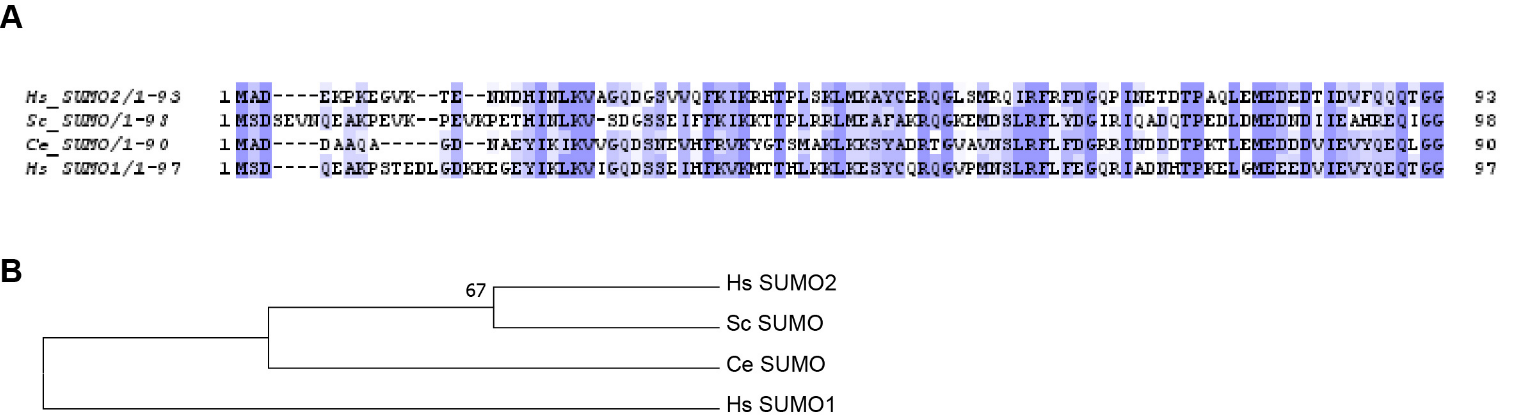
A
Hs_SUMO2/1-93
Sc_SUMO/1-98
Ce_SUMO/1-90
Hs_SUMO1/1-97
B
67
Hs SUMO2
Sc SUMO
Ce SUMO
Hs SUMO1
